# Improving Short Term Clock Prediction for BDS-2 Real-Time Precise Point Positioning

**DOI:** 10.3390/s19122762

**Published:** 2019-06-19

**Authors:** Lina He, Hairui Zhou, Yuanlan Wen, Xiufeng He

**Affiliations:** 1 School of Earth Sciences and Engineering, Hohai University, Nanjing 210098, China; wwwgwyl@126.com (Y.W.); xfhe@hhu.edu.cn (X.H.); 2 German Research Centre for Geosciences, GFZ, 14473 Potsdam, Germany; 3 28th Institute, China Electronics Technology Group Corporation, Nanjing 210007, China; zhou_hairui@126.com

**Keywords:** BeiDou satellite navigation system (BDS), satellite clock prediction, generalized regression neural network, precise point positioning

## Abstract

Although there are already several real-time precise positioning service providers, unfortunately, not all users can use the correction information due to either cost of the service and limitation of their equipment or out of the service coverage. An alternative way is to enhance the accuracy of the predicted satellite clocks for precise real-time positioning. Based on the study of existing prediction models, an improved model combing the spectrum analysis (SA) and the generalized regression neural network (GRNN) model is proposed especially for BeiDou satellite navigation system (BDS)-2 satellites. The periodic terms and GRNN-related parameters including length and interval of sample data, as well as a smooth factor, are optimized satellite by satellite to consider satellite-specific characteristics for all the fourteen BDS-2 satellites. The improved model is validated by comparing the predicted clocks of existing models and the improved model with precisely estimated ones. The bias of the predicted clock is within ±0.5 ns over three hours and better than that of the other models and can be used for several real-time precise applications. The clock prediction is further evaluated by applying clock corrections to precise point positioning (PPP) in both static and kinematic mode for eight IGS (International GNSS Service) MGEX (Multi-GNSS Experiment) stations in the Asia-Pacific region. In the static PPP, the improved model is validated to be effective, and position accuracies of some IGS MGEX stations achieve more than 30.0% improvements on average for each component, which enables us to obtain sub-decimeter positioning. In the kinematic PPP, the improved model performs much better than the others in terms of both the convergence time and the position accuracy. The convergence time can be shortened from 1–2 h to 0.5–1 h, while the position accuracy is enhanced by 15.4%, 21.6% and 19.3% on average in east, north and up component, respectively.

## 1. Introduction

The performance of the Global Navigation Satellite System (GNSS) positioning depends on the accuracies of satellite orbit and clock products [1,2]. Thanks to the great effort of the International GNSS Service, for example, the Multi-GNSS Experiment project, several types of precise orbit and clock products for GNSS satellites including GPS (Global Positioning System), GLONASS, Galileo, BeiDou and more are provided in routine base with different latencies and accuracies to meet various positioning demands [3,4,5,6,7,8]. Among these products, the ultra-rapid product is generated for possible real-time applications by predicting orbits and clocks over the next 24 h. However, the precision of the predicted clocks cannot satisfy the requirement of real-time precise point positioning (RTPPP) although the orbit uncertainty of about 5–10 cm can be neglected [9]. With the growth of GNSS high precision applications, there are already a number of RTPPP providers, such as GFZ (German Research Centre for Geosciences) and Wuhan University. Unfortunately, not all users can use the correction information due to either cost of the service or the user location is not within the coverage area of the service. Therefore, to improve the accuracy of predicted satellite clocks, especially over short-term, is of considerable interest to make RTPPP reliable in the abovementioned circumstances [10,11].

Generally, clock prediction is firstly required for generating clock information for broadcast ephemeris. It is an indispensable GNSS function in order to provide standard positioning service and the prediction time could be several hours to a few days varying from system to system. The positioning performance depends heavily on the accuracy of the predicted clocks. Usually, a low-order polynomial model is employed, and the accuracy is about 0.53 ns and 0.64 ns for example for GPS and BeiDou satellite navigation system (BDS), respectively [12]. Higher accuracy is quite challenging for the prediction process, as the intrinsic physical characteristic of clocks is very complex and unstable also due to varying space environments [12,13,14]. As a result of these factors, usually clock offsets, including clock bias, frequency bias, and drift, contain strong non-linear and stochastic features which make the short-term precise modeling and prediction rather difficult [13,15]. Despite such difficulties, numerous studies were carried out, and several models were established for clock prediction, e.g., quadratic polynomial (QP) model, spectrum analysis (SA) model, Kalman filter (KF) and neural network (NN) model [16,17]. 

The widely used prediction models include first and second order polynomial models, for example, and the predicted clock in GPS broadcast ephemeris [18,19] are well studied and discussed [20]. However, it is certificated that the clock error of the quadratic polynomial model is accumulated rapidly with the predicted time increased [16,21]. Such a result was expected because the stochastic variations contained by satellite clock accumulated over time [20]. To improve the QP model, studies obtained four main periodic variances for GPS satellites based on the fast Fourier transform (FFT) method and analyzed the relationship between its amplitude and orbit period [22]. They show the incorporation of fixed-period harmonics, especially for the GPS satellite clocks, provides a very accurate predicting model. Even though the SA model with periodic terms included is an improvement to quadratic polynomial model, it is pointed out that the periodic function has to be determined reasonably by a rather long clock time series [14,17]. Meanwhile, Epstein et al. achieve an accuracy of 8 to 9 ns by predicting GPS clocks over six hours with a KF approach, but the estimation is not reliable due to occurring frequency jumps [23]. Moreover, artificial neural networks are used by scientists to predict clock offset [12,16,24,25], e.g., WNN (wavelet neural network) and BPNN (back propagation neural network), which has the ability to estimate non-linear time series by sample training. However, the topology structure of the WNN is hard to set up, while the BPNN needs an iterative procedure to determine the appropriate weights which is considerably time-consuming. 

While most of the abovementioned achievements focus on GPS clock, BDS clock attracts more and more attention. There are also three types of real-time clock products for BDS satellites, namely broadcast ephemeris clock products with the accuracy of about 6 ns, ultra-rapid products about 2–6 ns and real-time estimated corrections updated every 5–10 s [12,26]. After comparison of these products, it can be concluded that the ultra-rapid product plays an important role in decimeter-level real-time positioning services, which is made available by some analysis centers [12]. However, evident nonlinear system errors, i.e., periodic signals, are detected in ultra-rapid clock product [12,25]. To meet the requirement of highly precise applications, such as early warning and hydrographic surveying, an improved model is required to enhance its prediction. Regarding the benefit from artificial intelligence, GRNN (generalized regression neural network) is a generation of radial basis function networks and probabilistic neural networks proposed by Specht [27] and has a strong ability of generalization for non-linear and stochastic clocks. In this paper, an improved model based on the SA model and GRNN is developed to deal with BDS-2 satellite clock prediction.

In the following sections, the related typical models for BDS-2 satellite clock prediction are summarized in Section 2, including the quadratic polynomial model, spectrum analysis model and GRNN. Then, an improved model for BDS-2 satellite clock prediction is proposed, and satellite- specific optimal parameters are determined by experiments in Section 3. After preprocessing of clock offsets, clock accuracy, static and kinematic precise point positioning (PPP) are validated and analyzed in Section 4. Finally, Section 5 concludes with a discussion.

## 2. The Improved Model

For a better understanding of the improved model for clock prediction, existing methods are introduced first. Then the improved model is presented as a combination of the SA model and the GRNN in detail.

### 2.1. Quadratic Polynomial Model

According to the performance of the satellite clock, the systematic part of clock offset can be described by polynomial models of a different order. Taking GPS satellite as an example, a linear polynomial is usually applied, as the difference between predictions using first-order or second- order polynomial is only 0.1 ns over three hours [19]. Since the stability of the GPS clock is higher than that of BDS, the quadratic polynomial model is commonly adopted as a basic model for BDS [12,28], which is presented as,

(1) $$clk\left(t_{i}\right)=a_{0}+a_{1}\left(t_{i}-t_{0}\right)+a_{2}{\left(t_{i}-t_{0}\right)}^{2}+\varepsilon_{i}$$

where *a*_0_ is the clock bias correction coefficient, *a*_1_ is the clock drift correction coefficient, and *a*_2_ is clock drift rate correction coefficient. *t*_0_ is the reference epoch of clock offset series*. t_i_* and *clk*(*t_i_*) denote the i-th epoch and its corresponding clock offset. *ε_i_* is the noise. 

### 2.2. Spectrum Analysis Model

Taking a quadratic polynomial model as a basic function, periodic terms are added to describe the clock offset, called spectrum analysis model. Previous experiments show that two significant periodic terms, which may be mainly caused by orbital effects, are applicable for BDS-2 satellite clocks, hence, the function with periodic terms is expressed as [12],

(2) $$\begin{array}{l} clk\left(t_{i}\right)=a_{0}+a_{1}\left(t_{i}-t_{0}\right)+a_{2}{\left(t_{i}-t_{0}\right)}^{2}\\ +b_{1}\sin\left(2\pi f_{1}\left(t_{i}-t_{0}\right)\right)+c_{1}\cos\left(2\pi f_{1}\left(t_{i}-t_{0}\right)\right)+b_{2}\sin\left(2\pi f_{2}\left(t_{i}-t_{0}\right)\right)+c_{2}\cos\left(2\pi f_{2}\left(t_{i}-t_{0}\right)\right)+\varepsilon_{i} \end{array}$$

where *a*_0_ is the clock bias correction coefficient, *a*_1_ is the clock drift correction coefficient, and *a*_2_ is clock drift rate correction coefficient. *t*_0_ is the reference epoch of clock offset series. *t_i_* and *clk*(*t_i_*) denote the i-th epoch and its corresponding clock offset. *b*_1_*, c*_1_ are the coefficients for the main period 1/*f*_1_, of which amplitude is $\sqrt{b_{1}^{2}+c_{1}^{2}}$. *b*_2_*, c*_2_ are the coefficients for the secondary period 1/*f*_2_, of which amplitude is $\sqrt{b_{2}^{2}+c_{2}^{2}}$. *ε_i_* is the noise.

### 2.3. GRNN Model

**Figure 1:**
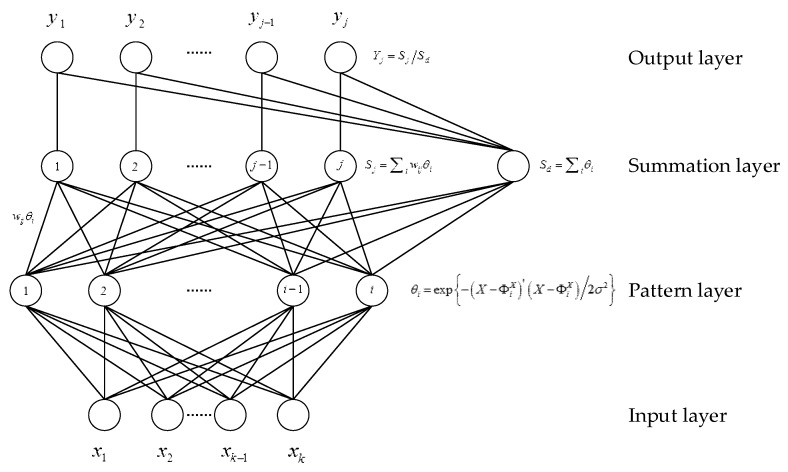
Basic architecture for generalized regression neural network (GRNN) [29].

GRNN is an improvement of radial basis function networks and probabilistic neural networks and has been applied in a variety of fields, such as time series prediction, system identification, and adaptive control. The basic network architecture of GRNN with multi-input and multi-output is shown in Figure 1 [29]. 

Figure 1 shows GRNN contains four layers, including input layer, pattern layer, summation layer, and output layer. The input layer provides the measurement variables to all of the neurons in the pattern layer, each pattern neuron represents a training pattern, and the output of each pattern neuron is a measure of the distance of inputs based on the stored pattern. The summation layer consists of two summation neuron types: One type computes the summation of the weighted outputs of the pattern layer, the other type calculates the un-weighted outputs of the pattern neurons. Finally, the output layer performs the normalization step to compute the GRNN predicted value of the output variable. 

As shown in Figure 1, *X* = [*x*_1_*, x*_2_, …, *x_k_*] is the input vector of the network and $\Phi_{i}^{X}$ is the training sample vector of the i-th neuron in the pattern layer. ${\left(X-\Phi_{i}^{X}\right)}^{\prime }\left(X-\Phi_{i}^{X}\right)$ is the squared distance between the input vector *X* and the i-th training sample vector $\Phi_{i}^{X}$. *σ* is a smooth factor that controls the estimated density. $\theta_{i}$ is the output of i-th neuron in the pattern layer. $w_{ij}$ is the connection weight between pattern layer neuron *i* and summation layer neuron *j*. The summation layer obtains the summation $S_{j}$ of the weighted outputs of the pattern layer and the un-weighted outputs $S_{d}$ of the pattern neurons. The output layer merely divides $S_{j}$ by $S_{d}$ to yield the desired estimate *Y* = [*y*_1_, *y*_2_, …, *y_j_*].

The smoothing parameter *σ* plays a key role in the GRNN model. As *σ* becomes very large, the estimated density is forced to be smooth, and GRNN model ignores detail characteristics of the training sample. With the decrease of *σ*, it is trying to track every characteristic of the training sample, which may lead to over-fitting with the loss of its generalization capability. Hence, a good choice of *σ* is necessary. A useful method of selecting *σ* is the holdout method with searching by experiments. For a particular value of *σ*, the holdout method removes one sample at a time and constructs a network based on all of the other samples. By repeating this for each sample and storing each estimate, the mean-squared error can be measured between the sample value and the estimate. The value of *σ* generating the smallest error should be employed in the final network. A detailed procedure for calculation of *σ* is described in [27].

### 2.4. The Improved Model

Since the stochastic characteristic of BDS-2 satellite clock is sophisticated and non-stationary, which cannot be described well using the existing models, an improved model combing the SA model and GRNN is proposed for short term clock prediction. It is known that GRNN has a high non-linear mapping ability and strong robust properties, which is suited for capturing the remaining clock residues after the removal of trend and periodic terms. Because there is no explicit expression for GRNN, the part of GRNN is depicted as a function of *f_GRNN_*. Hence, the improved model is proposed as,

(3) $$\begin{array}{l} clk\left(t_{i}\right)=a_{0}+a_{1}\left(t_{i}-t_{0}\right)+a_{2}{\left(t_{i}-t_{0}\right)}^{2}\\ +\sum_{m=1}^{2}\left[b_{m}\sin\left(2\pi f_{m}\left(t_{i}-t_{0}\right)\right)+c_{m}\cos\left(2\pi f_{m}\left(t_{i}-t_{0}\right)\right)\right]+f_{GRNN}+\varepsilon_{i} \end{array}$$

where *m* is the number of periodic terms obtained from SA model, and *b_m_* *, c_m_* are the coefficients for the period 1/*f_m_*. Other parameters are the same as Equation (2).

**Figure 2:**
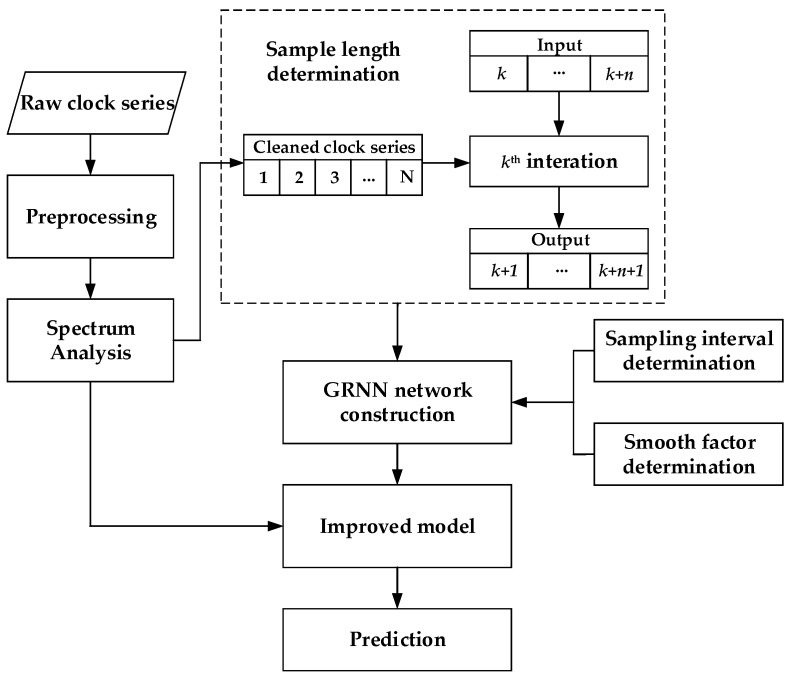
Data processing diagram for the improved model.

Further, the data preprocessing diagram is given in Figure 2. The input data set of the improved model are multi-days clock offset series. After preprocessing to get rid of outliers, a preliminary processed clock offset series is obtained. Then, the SA model is employed to weaken periodic characteristics, and the residuals are considered as input data set for GRNN. In the GRNN processing, different designs for GRNN parameters affect the prediction results very much. Therefore, as shown in Section 3, GRNN related parameters including length and interval of sample data as well as a smooth factor are optimized satellite by satellite to consider satellite-specific characteristics for all BDS-2 satellites.

## 3. Realization of the Improved Model

### 3.1. Preprocessing for Satellite Clock Offset

**Figure 3:**
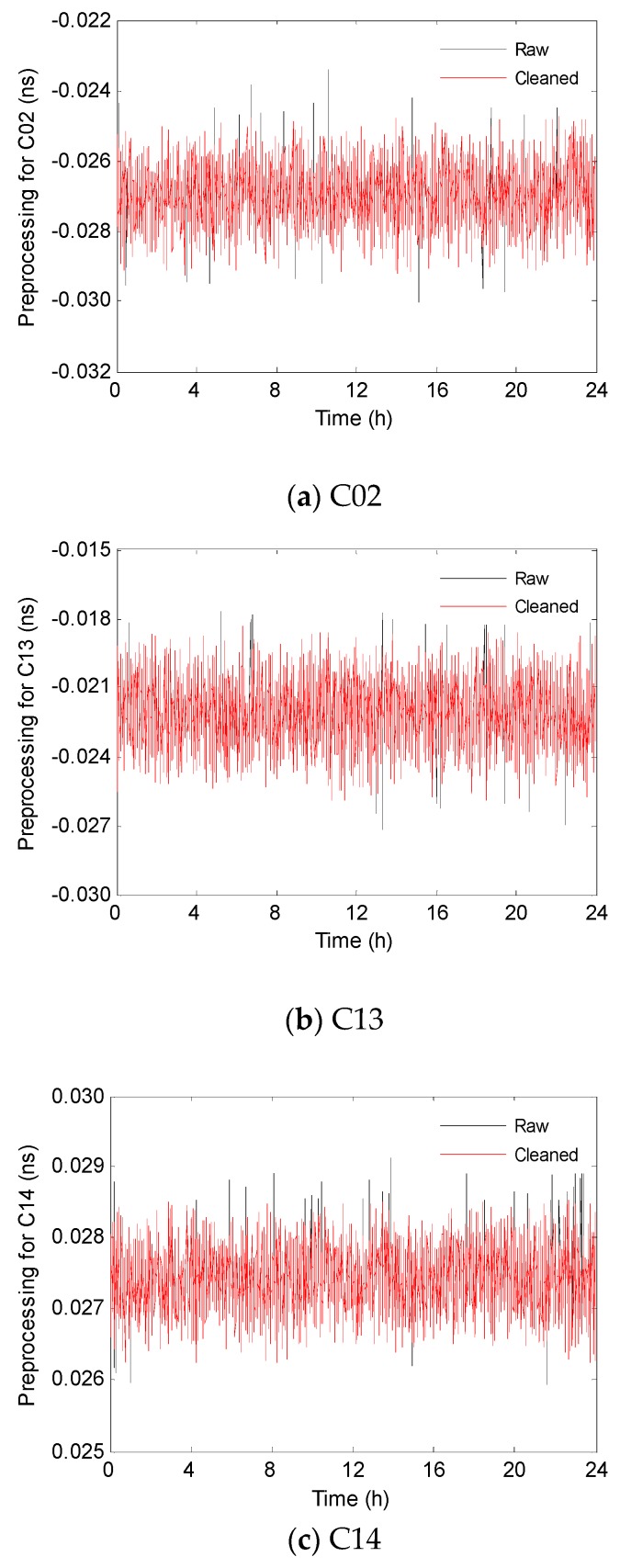
Preprocessing for satellite clock offsets. The raw data series are presented in black, while a cleaned time series getting rid of abnormal data are in red.

The estimated clocks of ultra-rapid products of BDS-2 which are made available by several GNSS analysis centers, for example GFZ, are used as input in data processing. However, the raw clock time series may conceal abnormal data, such as gross errors and blunders, which should be detected before performing clock prediction. The epoch-differenced clocks are calculated in which outliers can be shown more apparently and then the outliers are detected using the median absolute deviation (MAD), which is a robust but simple method [30]. In addition, the principle not to delete outliers more than 5% of total data is followed here, which avoids losing too much useful information [25]. Now, this preprocessing method is applied for each BDS-2 satellite using ultra-rapid products of GFZ with 5 min sample interval. Considering satellite clock stability is inversely proportional to its operation time, the newest launched satellites C02, C13, and C14, belong to GEO (Geostationary Earth Orbit), IGSO (Geosynchronous Satellite Orbit), and MEO (Medium Earth Orbit), respectively, are selected as examples to show the effectiveness of the MAD method in Figure 3. 

### 3.2. Determination of Periodic Terms

**Figure 4:**
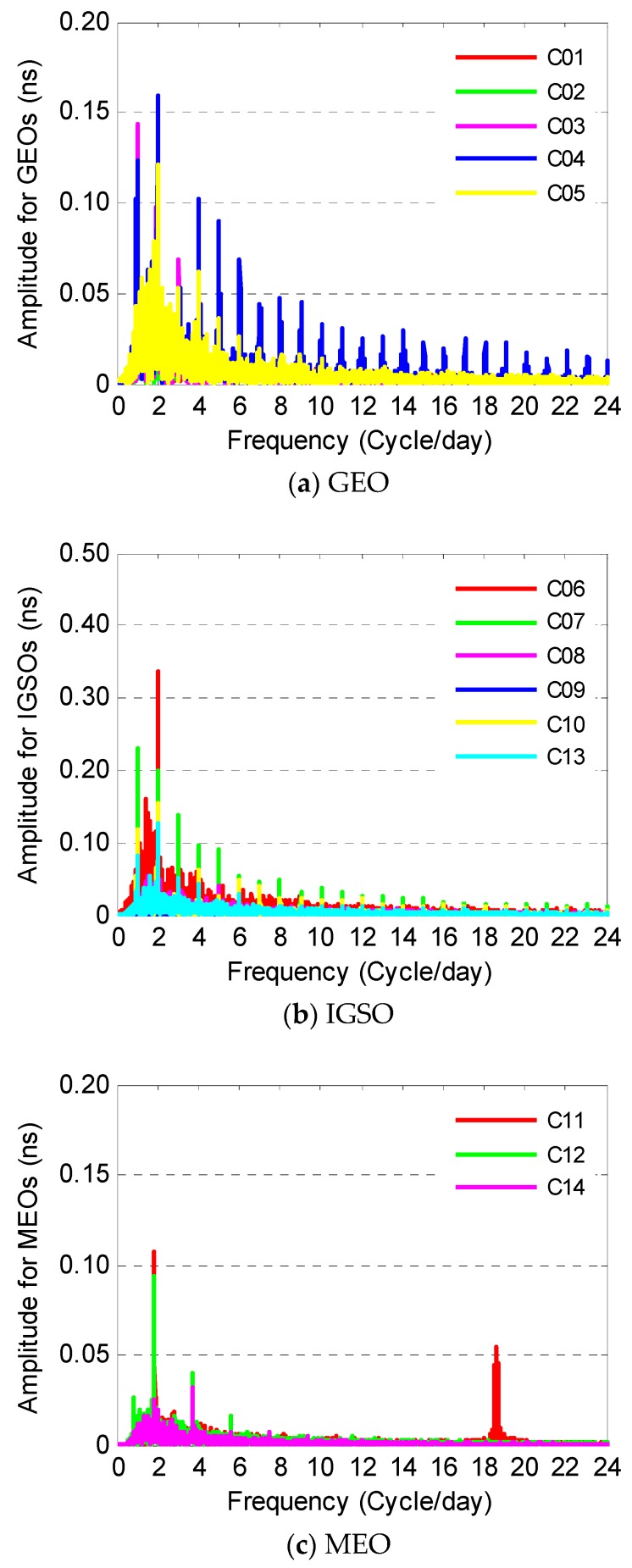
Frequency analysis for BeiDou satellite navigation system (BDS)-2 clocks.

Huang indicates that significant periodic characteristics are found in BDS-2 satellite clock residuals after quadratic polynomial fitting and periodic terms vary from satellite to satellite due to different geometric configurations [12]. In order to find out the potential periods for each BDS-2 satellite clock, FFT is applied to analyze the clock residuals after quadratic polynomial fitting using more than 100 days data from day 001 to day 105 in 2018. The relationships between amplitude and frequency are identified for GEOs, IGSOs, and MEOs, respectively in Figure 4, and the outstanding periods of each BDS-2 satellite clock are given in Table 1. Results show 24 h and 12 h are two significant periodic terms for most of GEOs and IGSOs with different amplitudes. As for MEOs, a 12 h harmonic with a smaller amplitude than for GEOs and IGSOs is noticeable. In general, the most noticeable periods of BDS-2 satellite clocks are related to their corresponding orbit periods. Interestingly enough, a special periodic term of about 1.3 h has been detected with a pronounced amplitude for the C11 satellite, and it is different from the normal period of the MEO orbital geometric configuration. This obvious period will have a large impact on prediction accuracy and must be considered. This noticeable period may be caused by the hardware of the atomic clock itself. Additionally, due to the uneven distribution of ground tracking stations and the relatively stable geometry between the GEO satellites and stations, the amount of data of C04 is less than other satellites, which is probably the reason that the residual noise level is large, and it leads to a saw-tooth pattern when doing FFT.

### 3.3. Impact of the Length of Input Data

The performance of the GRNN network differs in different sample datasets. Meanwhile, to build a suitable GRNN network, a sample dataset is required to be divided into input data and output data in an appropriate way. In this experiment, the remaining clock residues after the removal of trend and periodic terms are used, including all fourteen BDS-2 satellites. In the GRNN network construction phase, the length of input data successively increases from 1 h to 9 h to investigate how the length of input data affects the GRNN network performance. As an example, the length of input data is 5 h, in the first iteration, the GRNN network is constructed based on data of 1–5 h as input and with data 2–6 h as output. By analogy, in the second iteration, the GRNN network is optimized based on data of 2–6 h as input and with data 3–7 h as output. After completion of the (N-5)-th iteration (assuming there are N hour data), the GRNN network is employed for prediction.

**Figure 5:**
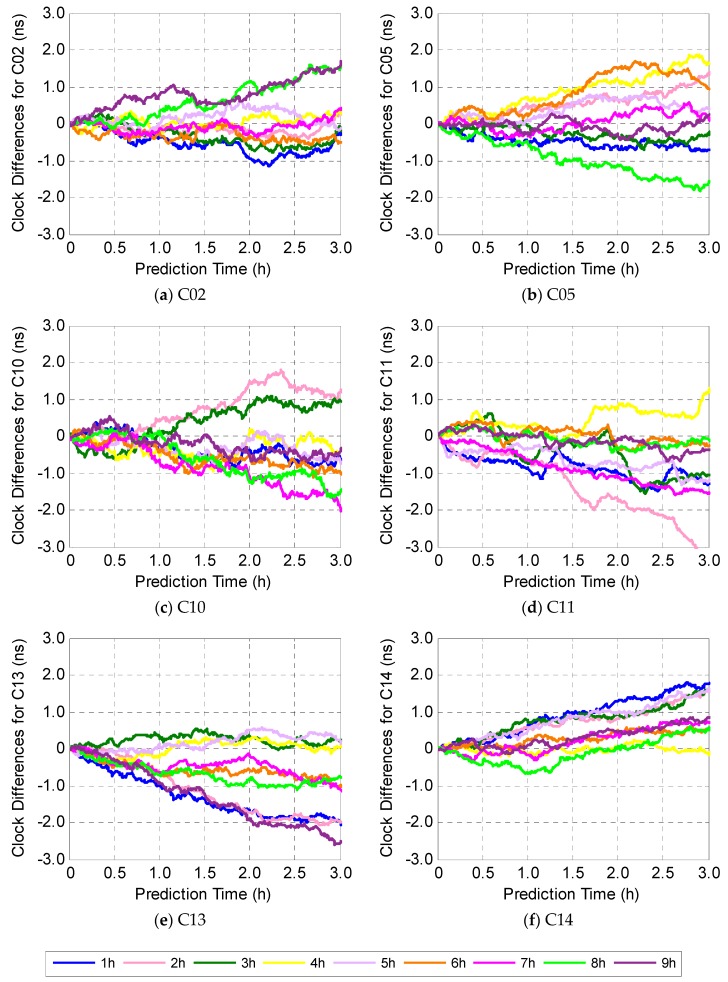
Clock differences between true (German Research Centre for Geosciences (GFZ) precise clock products) and prediction, using different lengths of input data.

Now, we can judge which sample length is the best for each BDS-2 satellite, including 5 GEOs, 6 IGSOs, and 3 MEOs, by checking the agreement of true (GFZ precise clock products) and prediction with different length of input data. Figure 5 gives clock difference results for each satellite type, and they are on the sub-nanosecond level when the prediction time is less than 1 h, no matter the length of input data raises from 1 h to 9 h. The clock differences for all fourteen BDS-2 satellites increase along with prediction time increasing, but are still within ±2 ns for most satellites, when the prediction time is not more than 3 h. Figure 5 also shows when a short length of input data is employed, i.e., 1–2 h, the characteristics of the satellite clock cannot be fitted properly. On the other hand, when the length of input data is up to 8–9 h, the clock differences are not the smallest, since too much perturbation information is introduced. After numerous experiments, when the prediction time is less than 3 h, the recommended length of input data is 3 h for most satellites as the best compromise between prediction accuracy and computational complexity. Additionally, it should be noted that the clock differences for all satellite clocks start from zero since the fitting ability of the GRNN is extremely strong and the training set and the test set are continuous.

### 3.4. Impact of the Sampling Interval of Input Data

Sampling interval reflects the intensive extent of input data, and it is another key parameter in a GRNN network. If the sampling interval is very small, a larger quantity of data joins in the GRNN network construction, and usually, it will lead to better predictions. However, the computation complexity increases dramatically with the number of neurons raising rapidly. With increasing sampling interval, sparse data may cause loss of short-term characteristics and decreasing the prediction accuracy. Therefore, sampling interval selection is worth to investigate for a better balance.

**Figure 6:**
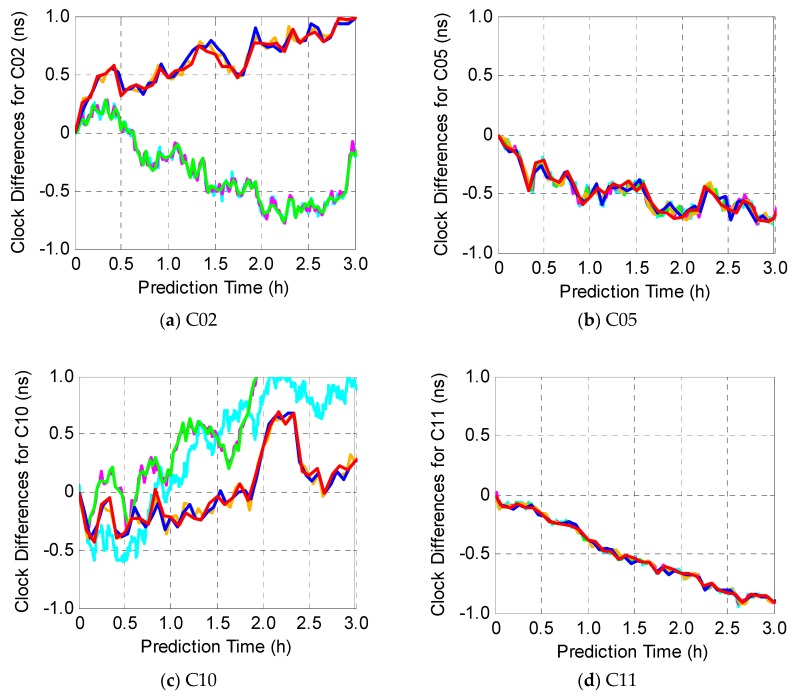

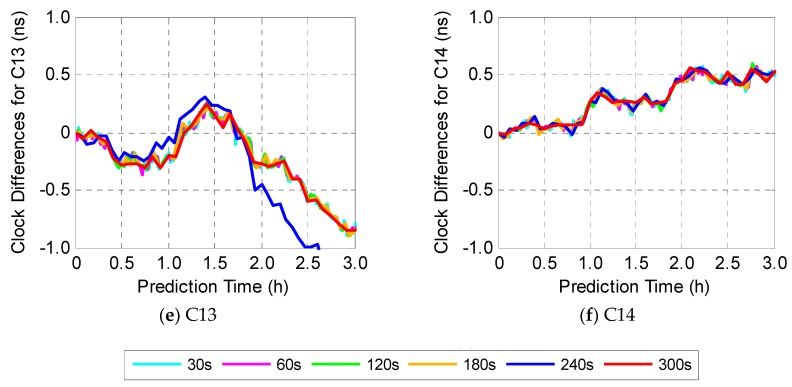
Clock differences between true (GFZ precise clock products) and prediction, using different sampling interval of input data.

In this experiment, the K-fold cross method is employed to investigate how the sampling interval affects the prediction accuracy, and therefore, the sampling interval of input data is set up to 30 s, 60 s, 120 s, 180 s, 240 s, and 300 s, respectively. The clock differences between true and prediction for two satellites of each type are presented in Figure 6. Overall, the sampling interval, less than 300 s, has little effect on the prediction accuracy. Additionally, the clock differences increase along with prediction time increasing but are still within ±1 ns for all fourteen BDS-2 satellites. In order to reduce the computing load, 300 s is choosing as the sampling interval in accordance with IGS 5 min clock products.

### 3.5. Determination of the Smooth Factor

Unlike a back propagation neural network, connection weights of a GRNN network are determined when the sample dataset is given. The smooth factor is the only adjustable parameter in a GRNN network, and it is well known that the smooth factor controls the influence sphere of input data, which significantly affects the prediction accuracy. With increasing the value of the smooth factor, the prediction curve is much smoother and neglects details. When the smooth factor is smaller, the approaching performance is better, but at the expense of computing time and the problem of over-fitting. Hence, the smooth factor for a GRNN network is required to carefully determinate. 

**Figure 7:**
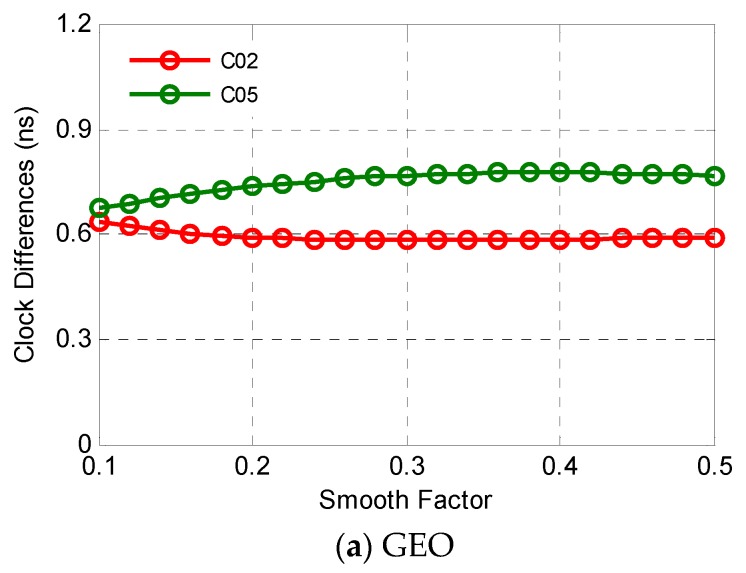

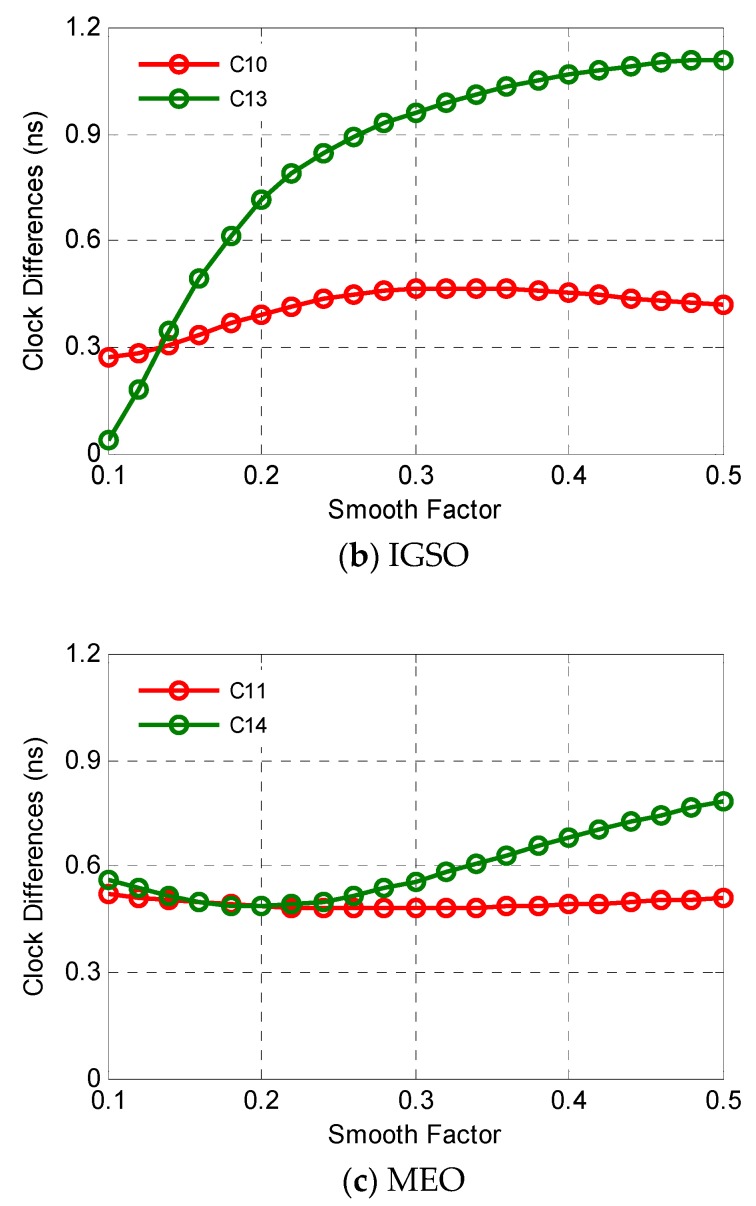
Clock differences between true (GFZ precise clock products) and prediction, using different smooth factors.

Since there is no a priori information, the smooth factor is determined by a search method in a special region. A series of values from 0.10 to 0.50 with steps of 0.02 is employed to check the prediction performance. Figure 7 indicates that the smooth factor should be satellite-specific determined, and the final results are given in Table 2. Taking C14 as an example, the smooth factor increases from 0.10 to 0.50, the clock differences between true and prediction firstly decrease and then increase with an optimal value of 0.20.

## 4. Validation of the Predicted Clocks

Generally, predicted clock accuracy can be evaluated by the deviation of prediction from its true value. However, the true value of the in-orbit clock is unknown. Therefore, a reference benchmark should be given. On the other hand, the predicted clock can be checked together with its related orbit for precise point positioning. In this paper, both evaluations are carried out, and the later one is more important, as it will give a preliminary assessment of the positioning accuracy which is the main goal of this research. 

### 4.1. Clock Comparison

**Figure 8:**
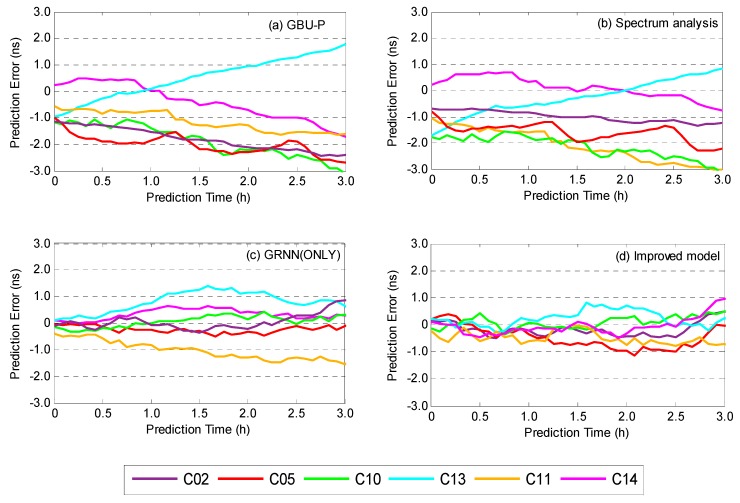
Clock differences between prediction and GFZ precise clock products, using (**a**) GBU-P, (**b**) spectrum analysis model, (**c**) GRNN (ONLY) model and (**d**) improved model.

First of all, GFZ precise clock products are selected as the reference benchmark to analyze the accuracy of clock prediction. To demonstrate the effectiveness of the improved model (combing SA and GRNN), other three predicted clocks are employed for comparison, i.e., GBU-P (it is a GFZ clock prediction product that uses QP model), spectrum analysis model, and GRNN(ONLY) model. Meanwhile, similar to the previous section, two latest launched satellites of each type are selected as examples to compare their prediction errors, as shown in Figure 8.

As shown in Figure 8, GBU-P and spectrum analysis prediction have very similar trends, and the spectrum analysis model performs better. Overall, their prediction errors are all within ±3 ns, when the prediction time is less than 3 h. The prediction errors of the GRNN (ONLY) model are smaller and more stable than the predictions utilizing the spectrum analysis, and the prediction errors of all satellites are keeping within ±1.5 ns. Comparatively speaking, the performance enhancement of the improved model is obvious and prediction accuracy has been increased. Whether the prediction time is 1 h or 3 h, the prediction errors of all satellites are within ±1 ns. Particularly, when the prediction time is less than 1.5 h, most of the prediction errors are within ±0.5 ns.

In order to further verify the prediction accuracy and stability of the improved model, 300-day clock predictions are evaluated statistically. The average clock differences between the predictions and GFZ precise clock products at different prediction time are calculated, and they are listed in Table 3, which also demonstrates that the improved model is evidently superior to the others. Compared with GBU-P, with the increase of prediction time from 0.5 h to 3.0 h, prediction errors of spectrum analysis are decreased by 3.6%, 6.6%, 12.6%, and 17.7% respectively. This shows that the short-term forecasting accuracy of GBU-P is acceptable. When the GRNN (ONLY) is used, maximum 72.2% improvement at 0.5 h and minimum 48.3% improvement at 3.0 h are obtained. When the improved model is employed, prediction errors are generally reduced by 70%, whether the prediction time is 0.5 h or 3.0 h. It demonstrates that the performance of the improved model has good stability.

### 4.2. PPP Validation

**Figure 9:**
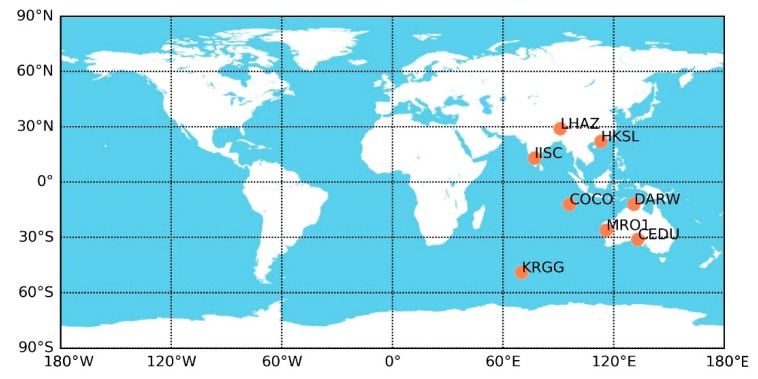
Tracking network for precise point positioning (PPP) validation.

Now, precise point positioning is carried out to further evaluate the capability of the improved model for BDS-2 satellites. Eight IGS MGEX stations, CEDU, COCO, DARW, HKSL, IISC, KRGG, LHAZ, and MRO1, in the Asian-Pacific region well covered by BDS-2 are selected and shown in Figure 9. The main processing strategies and parameters for both static and kinematic PPP are listed in Table 4, and the Position and Navigation Data Analysis (PANDA) software is applied for calculations [31,32].

#### 4.2.1. Static PPP Validation

Table 5 gives the position accuracy statistics of daily static PPP solutions in east, north and up components based on the IGS coordinates for the eight stations from day 001 to day 100 in 2018, using GBU-P, spectrum analysis, GRNN(ONLY) and improved model, respectively.

Compared with GBU-P, seven out of eight stations are enhanced using spectrum analysis model in the east component, and further optimized by GRNN(ONLY) model with the largest improvement of 29.3% at IISC station. Similar conclusions can be drawn in the north component for the first three methods. However, five stations perform better in the spectrum analysis and seven stations for GRNN(ONLY) model, compared with GBU-P. Meanwhile, accuracies of six stations’ coordinates in the up component have increased with the best of 28.7% at DARW station, using spectrum analysis model. When the GRNN(ONLY) model is used, maximum 38.2% improvement at LHAZ station and minimum 3.9% improvement at IISC station are obtained. In general, GBU-P can obtain an accuracy of decimeter level for most stations, using static PPP, while the sub-decimeter level is achieved for some special components for spectrum analysis and GRNN(ONLY) models.

When the improved model is employed, accuracies of all stations’ coordinates are enhanced further. Compared with GBU-P, the best performance in the east component is obtained at HKSL station, with an improvement of more than 40%. The corresponding improvement in the north component is 41.6% at LHAZ station. For the up component, the largest enhancement is 42.7% for LHAZ station. In all stations, the best improvement is visible at LHAZ station, accuracies are increased by 38.8%, 41.6%, 42.7% in east, north and up component, respectively. Generally, compared with GBU-P, the improved model achieves more than 30.0% improvements on average for each component. Additionally, more than 60% stations obtain sub-decimeter positioning accuracy at the horizontal direction.

#### 4.2.2. Kinematic PPP Validation

**Figure 10:**
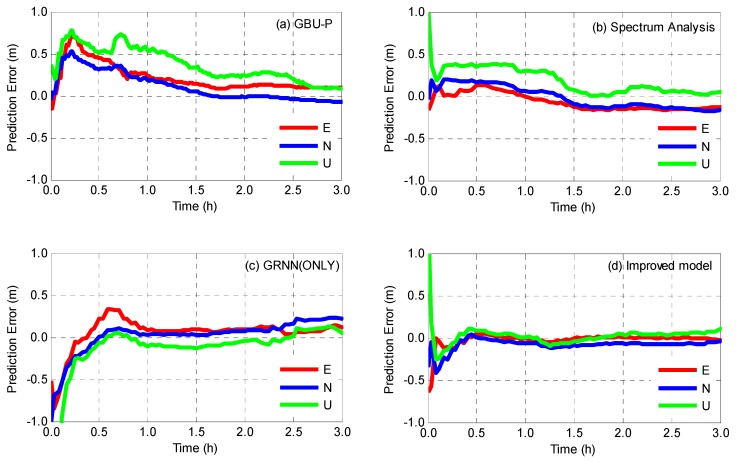
Position residuals of kinematic PPP for DARW station using (**a**) GBU-P, (**b**) spectrum analysis, (**c**) GRNN(ONLY) and (**d**) improved model, respectively.

**Figure 11:**
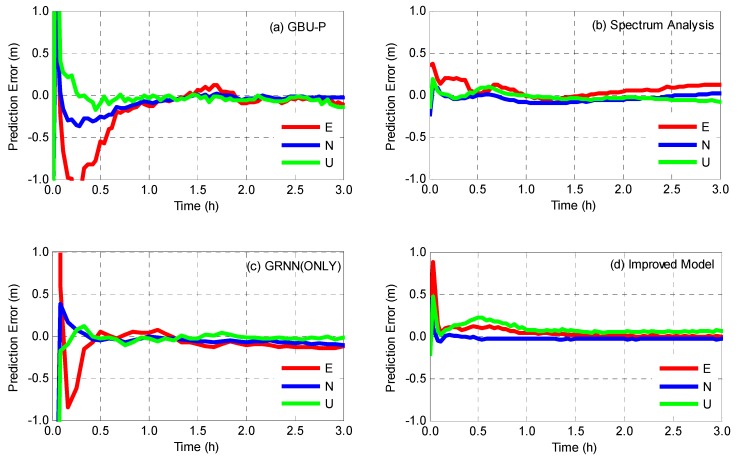
Position residuals of kinematic PPP for MRO1 station using (**a**) GBU-P, (**b**) spectrum analysis, (**c**) GRNN(ONLY) and (**d**) improved model, respectively.

The real-time kinematic PPP is realized for the abovementioned eight stations and position residuals of each station in east, north, and up component with respect to its IGS coordinates are calculated for evaluating the predicted clocks. Taking two stations DARW and MRO1 as examples, the time series of position residuals on day 005 is shown in Figure 10 and Figure 11, and the corresponding statistics of the two stations are listed in Table 6.

Figure 10 and Figure 11 intuitively demonstrate that the improved model provides better accuracy and shorter convergence time. As for GBU-P, position residuals converge after 1.5 h for DARW and 2 h for MRO1. Experiments results also show that the convergence time of the spectrum analysis model is shorter than that of GBU-P, and yet, GRNN achieves more stable position residuals for all three components after convergence. Comparatively speaking, position residuals obtained by the improved model converge rapidly within half an hour for DARW and one hour for MRO1 and keep within an accuracy level of ±0.1 m in the rest of the time. 

Table 6 shows that improved model has the best positioning accuracy. As for DARW station, compared with the GBU-P, the spectrum analysis model increases the position accuracy of 9.1%, 11.0%, and 13.1% for east, north and up component, respectively, while GRNN achieves 10.7%, 13.3%, and 19.3% improvement. When the improved model is used, positioning accuracies are increased by 14.5%, 25.2%, and 28.9% for DARW station (15.7%, 18.5% and 13.0% for MRO1 station) in east, north and up component, respectively. Statistical results also show that the improved model achieves 15.4%, 21.6%, and 19.3% improvement on average in east, north and up component, respectively.

## 5. Conclusions

Driven by requirements of real-time GNSS precise positioning, short-term precise clock predictions are improved for BDS-2 satellites for possible applications where real-time clock corrections are not available. An improved model combing spectrum analysis and GRNN is proposed. Further, the periodic terms and GRNN related parameters including length and sampling interval of input data, as well as a smooth factor are optimized satellite by satellite to consider satellite-specific characteristics for all the fourteen BDS-2 satellites. For example, there exist some special periods, e.g., 1.3 h for C11, beyond our understanding that periodic terms are related to the orbit period. This noticeable period may be caused by the hardware of the atomic clock itself.

The improved model with satellite-specific parameters is validated by comparing the predicted clocks of existing models and the improved model with precisely estimated ones. The clock accuracy of the improved model is generally better than that of the QP, SA, and GRNN model. The overall accuracy of predicted clocks is better than ±1 ns within 3 h, which is significantly enhanced with respect to the ultra-rapid products and the accuracy is good enough for several real-time precise applications.

The clock prediction is further evaluated by applying them to precise point positioning in both static and kinematic mode for eight IGS MGEX stations in the Asia-Pacific region. The position accuracies using predicted clocks obtained from QP, SA, GRNN models and the improved model are compared and their convergence times are also analyzed as well. In the static PPP, the improved model is validated to be effective, and helps to achieve more than 30.0% improvements on average for each component, which enables some stations to obtain sub-decimeter positioning accuracy. In the kinematic PPP, the improved model performs much better than the others in terms of both convergence time and position accuracy. The convergence time can be shortened from 1–2 h to 0.5–1 h, while the position accuracy is enhanced by 15.4%, 21.6%, and 19.3% on average in east, north and up component, respectively.

Additionally, compared to the polynomial model, the improved model needs extra processing time in the GRNN network construction phase. Supposing that *N* is the number of the whole data, *n* is the number of the input data in an iteration that is small enough to be ignored when compared with *N*, and *L* is the number of iterations. Therefore, the computational complexity of GRNN is *O(NL)*. However, the GRNN network construction is not a real-time task. Hence, the computing time of the improved model is almost at the same level as for the polynomial model in the clock prediction process. 

Furthermore, how to determine the smoothing factor value is a key issue. Generally, the Artificial Intelligence Community recommends the determination method of the smooth factor as the search method, which is an empirical method. A feasible way is determining the value of the smooth factor based on the relationship between the smooth factor and the clock quality determined by the Allan variance. However, it is a challenging topic to build function mapping between the smooth factor and the clock quality, and it is also an interesting topic, which will be investigated in our future work.

## Figures and Tables

**Table 1:** BDS-2 satellite clock periodic terms analysis.

Type	Satellite	Main Period (h)	Secondary Period (h)
GEO	C01	5.9850	24.0059
	C02	7.9727	12.0029
	C03	23.7449	11.8725
	C04	11.9373	23.2397
	C05	11.9700	5.9850
IGSO	C06	11.9373	23.6166
	C07	23.8747	11.9700
	C08	11.9700	23.8747
	C09	23.8747	11.9373
	C10	11.9373	23.8747
	C13	11.9700	23.8747
MEO	C11	12.8125	1.2922
	C12	12.8502	6.4157
	C14	6.4063	12.8125

**Table 2:** Recommended smooth factor for each BDS-2 satellite.

Type	Satellite	Smooth Factor
GEO	C01	0.10
	C02	0.26
	C03	0.10
	C04	0.10
	C05	0.10
IGSO	C06	0.10
	C07	0.50
	C08	0.38
	C09	0.10
	C10	0.10
	C13	0.10
MEO	C11	0.26
	C12	0.50
	C14	0.20

**Table 3:** Prediction errors at different prediction times.

Prediction Time (h)	GBU-P (ns)	SA (ns)^1^	GRNN (ns)	IM (ns) ^1^
0.5	1.541	1.486	0.429	0.412
1.0	1.631	1.523	0.513	0.437
2.0	2.226	1.946	0.915	0.505
3.0	3.051	2.512	1.578	0.841

^1^: SA means the spectrum analysis model, and IM means the improved model.

**Table 4:** Strategies and parameters for PPP validation.

Solutions	Setting
Software used	PANDA
Tracking stations	Eight stations in Asia-Pacific Area
Observations	Ionospheric-free linear combination
Sample rate	30 s
Elevation cutoff angle	7°
Weight	Elevation dependent
Orbit	GFZ ultra-rapid orbit
Trop zenith delay	Initial model (Global Mapping Function) and random walk
Phase windup	Phase windup correction
Earth tides correction	Solid
Earth Rotation Parameters	Products by GFZ
Differential code biases	Products by CODE
Receiver clock	White noise parameter
Ambiguity	Real constants
Parameter estimator	Least square method and Kalman Filter

**Table 5:** The standard deviation (STD) values of each station in North (N), East (E), and Up (U) directions of the daily static PPP solutions (one position solution for each 24-h interval).

Station	E (m)				N (m)				U (m)			
	GBU-P	SA^1^	GRNN	IM^1^	GBU-P	SA^1^	GRNN	IM^1^	GBU-P	SA^1^	GRNN	IM ^1^
**CEDU**	0.132	0.130	0.103	0.094	0.120	0.122	0.110	0.092	0.147	0.144	0.135	0.130
**COCO**	0.135	0.133	0.110	0.083	0.132	0.131	0.132	0.086	0.184	0.190	0.175	0.162
**DARW**	0.097	0.089	0.092	0.084	0.095	0.107	0.099	0.076	0.115	0.082	0.088	0.095
**HKSL**	0.152	0.153	0.122	0.092	0.150	0.132	0.132	0.114	0.192	0.139	0.140	0.120
**IISC**	0.188	0.173	0.133	0.116	0.122	0.113	0.111	0.083	0.181	0.185	0.174	0.116
**KRGG**	0.150	0.138	0.108	0.105	0.162	0.151	0.136	0.112	0.166	0.154	0.133	0.101
**LHAZ**	0.183	0.167	0.170	0.112	0.168	0.120	0.109	0.098	0.325	0.241	0.201	0.186
**MRO1**	0.132	0.125	0.118	0.098	0.120	0.121	0.094	0.088	0.172	0.146	0.133	0.135

^1^: SA means the spectrum analysis model, and IM means the improved model.

**Table 6:** The standard deviation (STD) values of DARW and MRO1 station in North (N), East (E), and Up (U) directions of the kinematic PPP solutions (after the initialization phase, with 5 min sample interval).

Method	DARW			MRO1		
	E (m)	N (m)	U (m)	E (m)	N (m)	U (m)
**GBU-P**	0.131	0.127	0.176	0.140	0.135	0.161
**SA ^1^**	0.119	0.113	0.153	0.129	0.118	0.153
**GRNN**	0.117	0.110	0.142	0.123	0.109	0.149
**IM ^1^**	0.112	0.095	0.125	0.118	0.110	0.140

^1^ SA means the spectrum analysis model, and IM means the improved model.
